# Broad Applications of Thiazole Orange in Fluorescent Sensing of Biomolecules and Ions

**DOI:** 10.3390/molecules26092828

**Published:** 2021-05-10

**Authors:** Ohad Suss, Leila Motiei, David Margulies

**Affiliations:** Department of Chemical and Structural Biology, Weizmann Institute of Science, Rehovot 7610001, Israel; ohad.suss@weizmann.ac.il (O.S.); leila.motiei@weizmann.ac.il (L.M.)

**Keywords:** fluorescent sensors, turn-on sensors, thiazole orange

## Abstract

Fluorescent sensing of biomolecules has served as a revolutionary tool for studying and better understanding various biological systems. Therefore, it has become increasingly important to identify fluorescent building blocks that can be easily converted into sensing probes, which can detect specific targets with increasing sensitivity and accuracy. Over the past 30 years, thiazole orange (TO) has garnered great attention due to its low fluorescence background signal and remarkable ‘turn-on’ fluorescence response, being controlled only by its intramolecular torsional movement. These features have led to the development of numerous molecular probes that apply TO in order to sense a variety of biomolecules and metal ions. Here, we highlight the tremendous progress made in the field of TO-based sensors and demonstrate the different strategies that have enabled TO to evolve into a versatile dye for monitoring a collection of biomolecules.

## 1. Introduction

The rise of fluorescence spectroscopy as a means to visualize biological systems at the molecular level has led to substantial findings about cellular and physiological mechanisms. Fluorescent probes have been heavily employed to monitor and report on a variety of processes such as the dynamics of cellular structures, protein expression and localization, enzymatic activity, and regulation of signaling networks [1,2]. Such probes can be introduced genetically by the expression of fluorescent proteins inside cells [3,4] or be added and taken up by cells from their environment [5,6,7,8,9,10]. In order to faithfully carry out their job, fluorescent probes must be highly sensitive and selective towards their target of interest, resulting in a specific fluorescent response. These requirements have pushed researchers to embrace more creative physical and chemical designs, prompting the development of ever more sophisticated fluorescent molecules that can reliably report on their surroundings.

Examples of such advancements are ‘turn-on’ or ‘turn-off’ fluorescent probes, which can change their fluorescent output after interacting with a desired target. With such probes, binding to the target either disrupts or initiates photophysical processes [11,12,13], such as Förster resonance energy transfer (FRET) [14], photo-induced electron transfer (PET) [15], or intramolecular charge transfer (ICT), which control the fluorescence of the reporter dyes. For example, ICT-based probes, which utilize solvatochromic dyes [16,17,18,19], change their emission intensity and fluorescence maxima in response to changes that occur in their environment’s polarity. Employing these mechanisms has enabled researchers to carefully fine-tune the response of probes while minimizing background signals that characterize traditional fluorescent dyes. Despite the progress provided by such sensory systems, ICT-, PET-, and FRET-based processes are not always strongly affected by analyte binding and their control over the dyes’ initial fluorescence is often limited, which can lead to the generation of high background signals.

**Figure 1:**
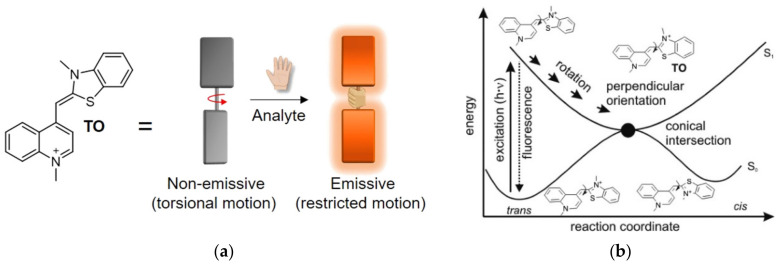
The turn-on fluorescence response of thiazole orange (TO). (**a**) The chemical structure of TO, along with a cartoon representation of its internal torsional movement (aromatic subunits are depicted by rotating plates), which upon restriction yields fluorescence; (**b**) Reaction coordinate of the fluorescence mechanism governing TO. Torsional motion around the methine bond of TO in the excited state (S_1_) leads to its depletion by non-radiative decay to the ground state (S_0_). However, restriction of this motion allows the excited state of TO to decay fluorescently. Reproduced with permission from [28]. Copyright 2016 American Chemical Society.

In an attempt to increase the sensitivity of turn-on fluorescent probes and to expand the mechanisms underlying their function, much attention has been devoted to the use of dyes governed by torsional motions as the candidates of choice for fluorescent sensing in biological environments [20,21]. This class of fluorescent dyes share a unique turn-on response mechanism, namely, their emission is enhanced once torsional motions around the bonds connecting their different segments are restricted (Figure 1a). A well-known group within this family of dyes are asymmetrical cyanines [22,23], which consist of two different heterocyclic subunits connected by one or several bridging methine bonds. In aqueous solutions, such dyes generally exhibit negligible fluorescence due to intramolecular twisting motions around their methine bonds, which result in a rapid non-radiative decay of the excited state. However, by hampering this motion due to increased viscosity, aggregation, or restrictive binding events, planarity is achieved between the twisting segments, which in turn leads to a markedly increased fluorescence. A well-studied member of this group is Thiazole Orange (TO, Figure 1a), consisting of conjugated benzothiazole and quinoline aromatic rings. TO, which was originally considered as a potential sensor for analyzing reticulocytes [24], is essentially dark in solution, yet it displays a thousand-fold increased fluorescence upon the introduction of double-stranded RNA and DNA (dsDNA or dsRNA). TO can intercalate or stack between base pairs and inside grooves of nucleic acids, leading to constricted torsional motion within the dye and increased fluorescence. The detailed physical mechanism underlying the fluorescent response of TO has been thoroughly studied and described before (Figure 1b) [25,26,27]. The high signal-to-noise (S/N) ratio displayed by this dye has prompted ever-growing interest in incorporating TO into fluorescent probes aimed at sensing biological targets.

Owing to TO’s proven interactions with RNA and DNA, the detection of nucleic acids has long been the focus of TO-based sensors. This has brought about various sophisticated molecular devices that, in some cases, can detect even a single mismatched mutation. Nevertheless, TO fluorescence has also been shown to be induced by several other binding events that do not involve intercalation into nucleic acids, for example, interactions with supramolecular hosts or amino acid side chains. This has enabled the implementation of TO in sensing a variety of other types of analytes, such as ions, small molecules, and proteins. This review summarizes the use of TO for the sensing of biomolecules and ions, as well as highlights notable examples of its application in the field.

## 2. Sensing of Nucleic Acids

**Figure 2:**
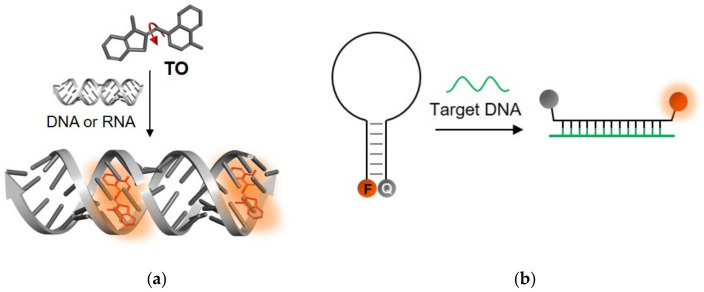
Comparison of nucleic acid recognition by TO and MBs. (**a**) TO’s ‘turn-on’ fluorescence results from its binding to dsDNA or dsRNA, regardless of the sequence or structure; (**b**) The operating mechanism of MBs relies on their embedded sequence, which allows for specific responses. In this illustration, recognition of a complementary target causes the hairpin structure to open up, separating the fluorophore (F) and quencher (Q) pair and yielding at ‘turn-on’ signal.

Nucleic acids are undoubtedly the most thoroughly described targets of TO-based sensors. The dye was first proposed for the detection of reticulocytes using flow cytometry [24], replacing weaker and less sensitive dyes such as acridine orange and thioflavin-T. In this pioneering work, TO was shown to be extremely sensitive to complexation with DNA; it increased its fluorescence by more than 3000-fold with a high S/N ratio. Additional studies [26] revealed that TO binds to double-stranded nucleic acids by either intercalating into their duplex structures (Figure 2a) or by sticking to their grooves; therefore, it comes as no surprise that TO has been implemented increasingly more as a universal staining dye in the gel electrophoresis of DNA and RNA [29,30,31].

Although fluorescent probes for sensing nucleic acids have been in use long before the discovery of TO, these systems suffer from several limitations that can reduce their detection accuracy. For example, fluorophores such as DAPI, ethidium bromide, and the Hoechst family of dyes are commonly used for staining nucleic acids in gels or for imaging cell nuclei [32,33]. However, these dyes are excited in the UV spectral region, which limits their use in various biological samples. First, UV light excites naturally emitting molecules, such as flavins and NADH, which results in the generation of high background fluorescence [34]. Second, prolonged exposure of cells to UV light, particularly of DNA and RNA within them, can inflict damage on the very molecules being investigated. These limitations have stimulated the development of probes that can emit at longer wavelengths and that exhibit improved S/N ratios, preferably through a ‘turn-on’-type mechanism.

A shared drawback of TO and other common dyes that are used in nucleic acid staining is their lack of structure or sequence specificity (Figure 2a). Currently, molecular beacons (MBs) [14,35], hairpin-like structures bearing a couple of FRET partners or a fluorophore-quencher pair on their stems, are perhaps the most prominent ‘turn-on’-type sensors for specific DNA and RNA detection (Figure 2b). In contrast to TO, these sensors undergo conformational changes in response to binding a complementary target sequence, leading to the opening of the beacon structure and to the dye pair moving apart and producing the desired signal. Despite their simple and efficient sensing mechanism and ability to detect specific RNA and DNA strands, MBs have several drawbacks. First, MBs generate relatively high background signals due to insufficient quenching of the reporter dye’s fluorescence. Non-specific interactions within complex environments such as inside living cells, or enzymatic degradation events, could lead to premature structural loss of MBs, leading to false-positive readouts. Finally, the dependence on FRET reduces the adequacy of MBs for detecting mismatched sequences, since they are less likely to be influenced by minute differences in target structures.

In comparison with MBs, TO exhibits a very low initial fluorescence and a substantial increase in its emission upon binding to nucleic acids, which does not involve FRET. These properties have made the dye a leading candidate for designing advanced, specific, and low-background molecular probes able to sense DNA and RNA. Careful chemical modifications of TO have enabled the emergence of sensors that can be applied for in vitro detection of nucleic acids and even for imaging of RNA targets in living cells and tissues. These sensors can be classified into three main subtypes: (a) sequence-targeting probes, (b) structure-targeting probes, and (c) probes that can detect protein–DNA interactions. The first subtype relates to molecular hybridization probes directed at specific sequences of DNA or RNA, commonly employing complementary sequences for a desired target. The second subtype includes probes that can principally interact with certain nucleic acid structures and conformations, either in a statistically favorable manner or by using stabilizing agents. The last subtype employs TO-labeled DNA-binding proteins to sense the formation of protein–DNA complexes.

### 2.1. Sequence-Targeting Probes

#### 2.1.1. Probes Appended with Terminal TO

**Figure 3:**
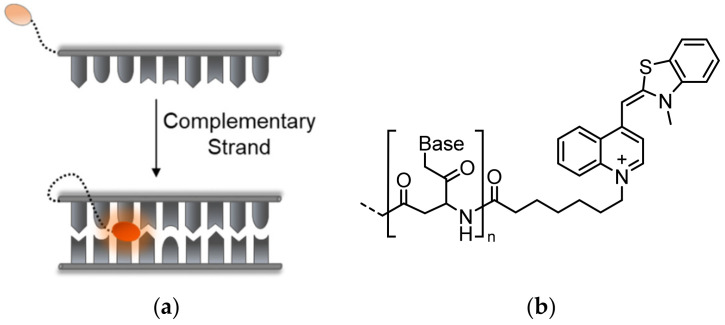
Light-Up probes. (**a**) The operating mechanism of Light-Up probes: upon hybridization to a target sequence, TO (orange oval) intercalates within the formed duplex and displays increased fluorescence; (**b**) the general chemical structure of PNA-based Light-Up probes.

Early attempts at designing TO-modified probes for sensing specific DNA sequences were carried out using peptide nucleic acid (PNA) chain-based hybridization modules, which were termed Light-Up probes (Figure 3a). Introduced by Kubista and his colleagues, Light-Up probes contain sequences of nucleic bases on a PNA backbone appended at their end with TO through a flexible linker (Figure 3b) [36,37]. Aside from providing resistance to degradation by nucleases, using a neutrally charged PNA backbone in place of a highly negatively charged DNA backbone minimizes background fluorescence by preventing intramolecular interactions between the positively charged TO and a DNA strand. Furthermore, the PNA-DNA complexes are more stable than dsDNA, allowing for better association and signal production. Upon introduction of complementary target strands, Light-Up probes respond with up to a 50-fold increase in fluorescence. This observation has made Light-Up probes convenient for detecting amplified DNA in PCR methods including real-time PCR [38,39]. TO-based Light-Up probes were also used as energy donors in a FRET-based system to sense RNA maturation and splicing [40], yielding increased energy transfer to a PNA acceptor. In another example, pseudo-complementary versions of Light-Up probes were used to detect specific DNA sequences and deliver site-specific photo-induced damage by creating reactive oxygen species [41].

Despite their success, Light-Up probes are generally limited by the relatively high background signal that they generate, which results from intramolecular interactions between the flexibly linked TO and the bases of the PNA probe [42]. This, along with some specificity issues when tested in the context of large DNA samples, causes detection by Light-Up probes to be susceptible to false positives. Moreover, changes in the probe’s length, sequence, and the type of linker used to attach TO to PNA all seem to unpredictably affect the probes’ quantum yields, requiring a case-by-case study of each probe prior to its use [28,43].

Dervan [44] and Asseline [45] tested TO-appended pyrrole-imidazole (Py-Im) polyamides and pyrimidine chains, respectively, as an alternative to PNA-based Light-Up probes. Depending on their comprising subunits, these conjugates, although lacking nucleic bases, can target specific sequences of dsDNA to form triplexes. The Py-Im probes exhibited improved S/N ratios and a near 1000-fold increase in fluorescence upon interaction with their targets. However, despite Py-Im sensors being well reviewed as promising DNA sensors [46,47], research on TO-modified versions is very limited.

#### 2.1.2. Probes with Internally Embedded TO

##### PNA-Based Probes

**Figure 4:**
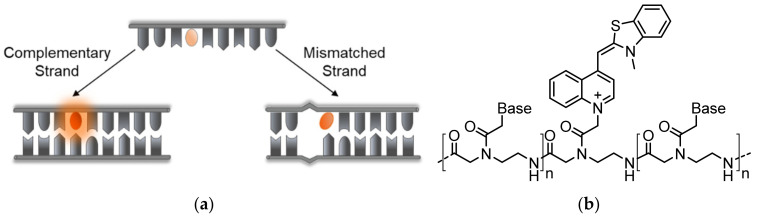
PNA-based FIT-probes. (**a**) The operating mechanism of FIT-probes: an internally embedded TO is forced to intercalate within a specific location of a formed duplex, thereby making it sensitive to local mismatched bases that affect its motion restriction and the resulting fluorescence; (**b**) The chemical structure of TO-based PNA FIT-probe.

In an effort to prevent non-specific intramolecular activation of TO and to improve probe sensitivity, Seitz and his team established TO as a broad-use base surrogate that can be inserted within the probe’s sequence (Figure 4a). TO was connected to a PNA backbone by a short carboxymethylene linker, similar in length to the linkages of PNA and DNA bases, tethered to its quinoline ring (Figure 4b) [48,49]. Compared to Light-Up probes, in which flexibly linked TO can interact with a target in various ways, the new probes enforce the intercalation of TO into a formed PNA complex with DNA or RNA at a predetermined location. These probes were therefore termed Forced Intercalation Probes, or FIT-probes. In the presence of complementary strands, the fluorescence of FIT-probes increases, with reported maximal values ranging from 20- to 30-fold [48,50]. Interestingly, the site-specific intercalation mode of TO, as part of the sequence of FIT-probes, causes these probes to display sensitivity towards single mismatches. FIT-probes presented with DNA targets containing mismatching bases adjacent to the TO intercalation site exhibited lower fluorescence than FIT-probes subjected to fully complementary strands. Presumably, mismatches allow for more torsional space for TO, lessening its restriction and therefore its quantum yield. This feature was also tested under real-time PCR and in qPCR experiments, demonstrating these probes’ ability to detect complementary RNA while simultaneously filtering out mismatched strands [51,52,53,54].

It should be noted that the absorbance and quantum yield of TO in PNA-based FIT-probes can be affected by the neighboring base pairs in the formed complexes [50]. Generally, bordering guanines act in quenching TO by energy transfer from its excited state. Time-resolved fluorescence assays have been performed to compensate for such effects, and to further elucidate the nature of interactions between FIT-probes and DNA. However, considering the overall ability of FIT-probes to sense their targets of interest, this issue seems to be minor.

A great attribute of PNA FIT-probes is their utility in imaging RNA in live cells. Using a specific probe against the viral mRNA of influenza H1N1, Seitz [55] was able to selectively detect the target in infected cells, resulting in a near 5-fold higher fluorescence than in non-infected cells. A later attempt expanded this system to dual-color imaging by adding a second FIT-probe, modified with a closely related benzothiazole orange (BO) dye that is specific towards the neuraminidase mRNA of H1N1 [53]. In a study by Gait and Seitz [56], FIT-probes were used to image the cellular localization of miRNA 122 in liver cells and to identify inhibitors of its activity. Wickstrom [57] and Yavin [58] made extensive efforts to employ FIT-probes for sensing of *K-ras* mRNA in cancer cells. Both studies achieved impressive results with single-nucleotide resolution, enabling researchers to distinguish between cells expressing the wild-type sequence and cells expressing a SNP mutation. In another study, Yavin was able to use FIT-probes as a diagnostic tool for sensing the high expression of the long non-coding RNA (lncRNA) cancer-associated transcript 1 (CCAT1) [59]. Notably, targeted sensing of CCAT1 was performed in colorectal biopsies, demonstrating the efficiency of PNA FIT-probes even in complex biological samples.

Despite their improved qualities, PNA-based FIT-probes still have a few drawbacks. Due to their large size, PNA FIT-probes often require cell permeabilization and delivery agents for their introduction into cells. As many studies acknowledge, the administration method chosen for FIT-probes can dramatically affect their resulting signal; thus, this must be considered when testing them in a biological context. Additionally, though quite simple and straightforward to synthesize, PNA FIT-probes diverge from native nucleic acids due to their neutral backbone and their increased hydrophobicity. This difference makes them prone to aggregation and, to a degree, unspecific binding. Solutions addressing these issues have been proposed and reviewed in detail [28,60,61,62].

**Figure 5:**
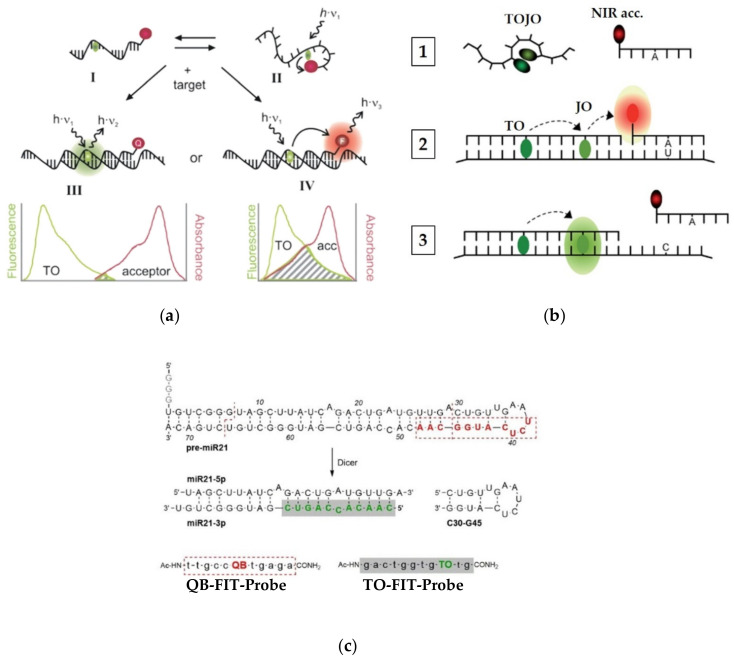
Multicolor PNA-based FIT-probes. (**a**) In the absence of a target, the initial fluorescence of TO (in green) is reduced by collisional quenching (I↔II) with an acceptor dye (in red, acting as either a quencher or FRET partner). The fluorescence is regenerated upon target recognition (III), which could also be accompanied by a FRET response (IV). Adapted with permission from [63]. Copyright 2012 Royal Society of Chemistry. (**b**) A multicolor FIT-probe FRET-based system for the sensing of C to U editing in RNA. In the absence of target RNA, a binary TOJO-labeled FIT-probe is dark due to formation of H-aggregates between the dyes (1). Recognition of an edited target yields hybridization of the binary probe, along with an acceptor NIR-labeled reporting strand, conferring FRET to the acceptor dye (2). Unedited RNA cannot bind the reporting strand; therefore, displaying only increased fluorescence from FRET to the JO dye on the binary probe (3). Adapted with permission from [64]. Copyright 2018 Royal Society of Chemistry. (**c**) Maturation of miRNA21 by dicer cleavage, and its targeting by TO and QB-labeled FIT-probes. Adapted with permission from [65]. Copyright 2020 John Wiley & Sons, Inc.

To further enhance the S/N ratio provided by FIT-probes, these TO-based systems have been also used as gateway donors in multicolor probes (Figure 5a). By positioning TO at a certain distance from a FRET acceptor dye, Seitz [63] was able to further reduce the minor initial fluorescence of PNA FIT-probes by one of two mechanisms. The first mechanism (Figure 5a, III) involves a negligibly-overlapping dye, placed 10 or more nucleotides away from TO, that physically collides with TO, to quench its emission. For example, indotricarbocyanine (ITCC) lowered the background signal of TO by over 99%, allowing for a signal increase of 450-fold upon target hybridization. The second mechanism (Figure 5a, IV) makes use of a FRET acceptor that is placed 4–10 nucleotides away from TO. The acceptor’s fluorescence in such probes is very low on its own due to depletion of TO’s excited state in the single strand; however, it increases dramatically once a target is introduced.

Seitz and his team [64] have explored multicolor PNA FIT-probes in sensing single nucleotide alterations occurring in living cells. In order to detect C to U editing of mRNA, Seitz combined a dual-color FIT-probe with a second reporting unit consisting of an acceptor-labeled strand to afford a multicolor FRET-based sensor (Figure 5b). The dual-color FIT-probe, which is complementary to a mRNA sequence of the human glycine receptor, contains TO and an oxazolopyridine analogue, JO, which together act as a turn-on FRET donor and a turn-on FRET acceptor, respectively. The two dyes can interact in the single-stranded probe to form H-aggregates that quench the fluorescence of both of them (Figure 5b, 1). A reporting strand, labeled with a NIR acceptor dye, is complementary only to an edited segment of the same mRNA. In the presence of the mRNA target, hybridization of TOJO resolves the H-aggregates, enabling bright fluorescence of JO through FRET with TO (Figure 5b, 3). Only the fully complementary edited version of the mRNA allows for strong binding of the NIR acceptor-appended strand (Figure 5b, 2), increasing FRET efficiency by a factor of 14 when compared to the unedited version. However, when examined in cells expressing the two versions of the target mRNA, the difference in FRET efficiency was more modest.

Efforts by the same group have also focused on tracking RNA editing from the angle of miRNA maturation (Figure 5c) [65]. Cleavage of a hairpin module of the premature miRNA-21 (pre-miR21) by the Dicer enzyme could be monitored by two carefully designed FIT-probes appended with distinct dyes, TO and quinoline blue (QB). The TO-FIT-probe binds the mature miRNA, whereas the QB-FIT-probe is targeted to a duplex-hairpin junction of the premature pre-miR21, exhibiting a 14-fold fluorescent increase upon its hybridization. Once pre-miR21 is excised at the target sequence of the QB-FIT-probe, its fluorescence is lowered significantly, as fluorescence of the TO-FIT-probe increases due to its recognition of mature miRNA. This property was further used to detect a component of the RNA silencing system, which, upon addition, allows for stronger hybridization of the TO-FIT-probe and a higher fluorescence increase. Therefore, the shifting QB/TO fluorescence ratio can be used to observe multiple ongoing enzymatic events that, in the future, might be translated into cell biological research.

##### DNA-Based Probes

Despite the various advantages of using PNA, the innate differences in the chemical properties of PNA-based probes from those of normal DNA and RNA may complicate detecting these targets in biological mixtures. As noted above, PNA-probes are more hydrophobic than DNA and RNA due to the lack of polar sugar-phosphate groups in their backbones. Consequently, this may lead to probes’ aggregation or cause some unspecific binding interactions. Thus, DNA-based TO-probes have been put forward as a means to more closely resemble natural nucleic acids. Compared to a PNA backbone, a DNA backbone is highly hydrophilic and hence, suitable for work in aqueous environments. On the other hand, its concentrated negative charge may lead to a high background signal owing to electrostatic interactions with the cationic TO dye. Moreover, a glycosidic bond between 2′-deoxyribose and a positively charged nitrogen of TO, as part of a surrogate base, would be very labile. As will be described hereafter, different strategies have been employed to solve such issues, yielding many types of DNA-based TO probes.

**Figure 6:**
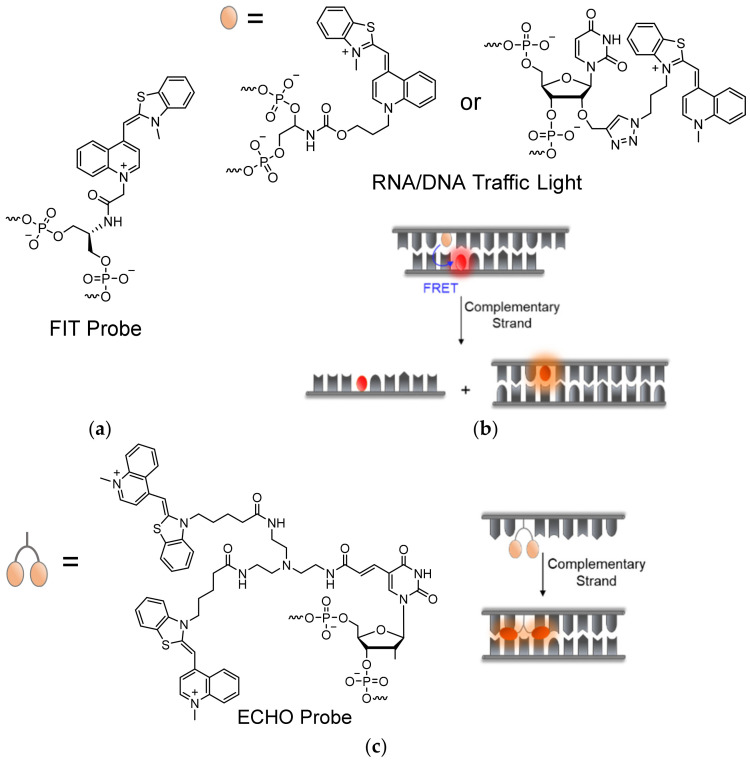
DNA-based TO probes. Presented are the chemical structures and the operating mechanisms of (**a**) DNA-based FIT-probes (refer to Figure 4a for the mechanism); (**b**) DNA/RNA traffic lights; (**c**) ECHO probes.

Expanding their nucleic acid-sensing arsenal, Seitz developed a new set of FIT-probes based on DNA (Figure 6a). In order to sterically hinder stacking interactions between TO and adjacent bases in the probe itself, while simultaneously conferring the necessary flexibility for target intercalation, short and rigid linkers were used, such as L-serinol and carbocyclic 2′-deoxyribose [66,67]. Indeed, these probes turned out to display higher fluorescence increases and better target specificity when compared to the PNA-based probes, manifested by their use in detecting RNA in PCR assays and cell lysates. The improved fluorescence intensities of DNA-based probes directly result from better fitting and intercalation of TO into double-stranded complexes with DNA or RNA. The efficiency of DNA FIT-probes was also enhanced by introducing an acceptor dye to TO, similarly to the previously described PNA multicolor FIT-probes (Figure 5). TOJO DNA-based FIT-probes were shown to have an improved S/N ratio, with the ability to visualize different developmental stages of drosophila oocytes by monitoring the localization of *oskar* mRNA in a wash-free manner [68]. The same biological model was later used to develop qFIT-probes for wash-free quantitative analysis of RNA in oocytes [69]. The qFIT-probes combine TO with Cy7, a NIR dye, positioned sufficiently far away from each other to prevent their interaction. In RNA imaging experiments, hybridized TO-activated qFIT-probes could be separated from non-hybridized probes and reveal mRNA enrichment in subcellular locations.

Wagenknecht demonstrated an alternative 5-atom linear carbamate-bonded linker for attaching TO to DNA-probes [70,71,72], and later through copper-catalyzed click chemistry to a 2′-*O*-propargylated uridine [73] or an arabino-configured analogue [74] (Figure 6b). Attachment of TO by site-specific click chemistry has the benefit of avoiding time-consuming preparation of TO-appended phosphoramidites, with the option of adding the dye in a post solid-phase stage of synthesis. Despite avoiding potentially labile glycosidic bonds as demonstrated in previous instances, some of these linkages afforded higher background fluorescence of non-hybridized probes due to induced stacking interactions with nearby bases. With this in mind, Wagenknecht and his team were able to utilize TO as a donor base, together with acceptor dyes in so-called DNA or RNA ‘traffic lights’ [72,75] (Figure 6b). The term ‘traffic lights’ refers to the FRET process between the green-emitting TO dye and a red-emitting Thiazole Red (TR) acceptor, originally introduced into this type of hairpin-like probes. Localization of TO and partner dyes opposite one another enables energy transfer or quenching interactions between them, which are interrupted upon target recognition due to separation between the TO-acceptor pair, thus yielding increased TO fluorescence [76]. Such probes were applied to determine the degree of delivery of siRNAs into cells, along with their inhibitory activity [75,77]. Subsequent works have broadened the library of clickable approaches for TO-labeling of DNA-based probes, combined with other acceptor dyes, through chemical modifications of thymine bases [78,79].

Studies of dimeric derivatives of TO have shown that excitonic interactions between the dyes act in quenching the fluorescence of the dimers, yielding a stronger response to DNA or RNA complexation and a higher S/N ratio [30]. This principle was employed by Okamoto in his development of exciton-controlled hybridization-sensitive oligonucleotides, or ECHO probes in short (Figure 6c) [80,81,82]. ECHO probes consist of two covalently linked TO units, connected by a flexible linker to the 5′-carbon position of a uridine base [83]. This structure promotes the formation of non-fluorescent dimeric TO H-aggregates, which, with the help of linker flexibility, dissociate upon hybridization to targets, increasing the fluorescence of the TO dyes. ECHO probes have been proposed for use as reporter primers for genotyping of SNP regions [84], and have been shown to be useful for RNA detection and imaging in live cells, bacteria, and complex tissue samples [85,86,87,88]. A shortcoming of ECHO probes is their non-specific binding to dsDNA, as well as to each other, due to the intrinsic affinity of the TO dimers to DNA [80,88,89]. Attempts to address such problems, however, involved the incorporation of unnatural bases that prevent cross-probe reaction [88], or the conjugation of the ECHO probes to bulky protein or nanoparticles to avoid diffusion of probes into specific cell organelles [90]. However, these approaches are limited in solving the issues described above.

Like PNA-based probes, DNA-based probes require special delivery methods when introduced into cells due to their sheer size and negative charge. These methods have been properly reviewed before [60]. Another issue that must be addressed when designing DNA-based probes is degradation by cellular nucleases. Degradation of DNA-based probes can lead to the release of reporter dyes into the cell, which either fail to produce a ‘turn-on’ signal completely or generate a high degree of false positive reads due to non-specific interactions. In order to decrease the vulnerability of DNA-based probes to nucleases, chemical modifications can be employed to prevent backbone recognition by the enzymes. One such modification is a 2′-*O*-MeRNA backbone, which was shown to protect ECHO probes from breakdown by nucleases and enabled long-term measurements in live cells [91,92]. Likewise, locked nucleic acid (LNA) backbones have been shown to provide synthetic DNA and RNA with nuclease resistance, as well as greater affinity towards complementary targets [93]. In LNA-probes, a cyclic deoxyribose unit is fixed by an intramolecular bridge in a C3′-*endo* pucker, which characterizes A-type conformations adopted by RNA in complex with DNA. It has been demonstrated that when positioned adjacently to a serinol-tethered TO, the LNA modification confers significantly higher quantum yields with a hybridized target and higher S/N ratios [94]. This is presumably achieved by the added rigidity at the TO hybridization site, lowering the chances of non-radiative decay. In ECHO probes, LNA was able to increase probe fluorescence and stability when introduced three to four bases away from the TO dimer [95]. By combining both LNA and 2′-*O*-MeRNA methods in a DNA-based probe bearing a QB dye in place of TO, Herrmann and Seitz [96] were able to selectively and sensitively detect the infection by influenza A virus in live cells.

### 2.2. Structure-Targeting Probes

**Figure 7:**
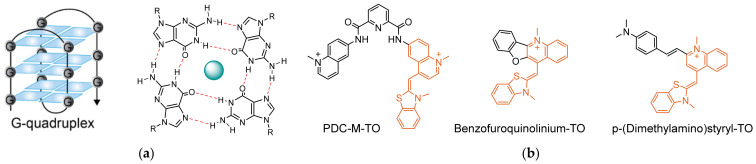
TO-based G-quadruplex-sensing probes. (**a**) Molecular structure and conformation of a representative G-quadruplex. G-tetrads are stacked parallel to one another with a stabilizing cation in the middle of each tetrad; (**b**) Examples of notable TO-based probes for selective sensing of G-quadruplexes.

Nucleic acids can adopt several secondary structures and conformations, depending on their sequence and environmental conditions, such as salt concentration, pH, and temperature [97,98]. Some of these structures have been found to be prevalent in biology, for example, DNA G-quadruplexes (Figure 7a) [99]. Formed by guanine-rich sequences, G-quadruplexes are characterized by tetrads of guanines arranged in stacked planar conformations through Hoogsteen hydrogen bonds that are stabilized by centrally positioned cations. G-quadruplexes are common in certain types of RNA, as well as in the telomeric ends of genomic DNA and the promoter regions of certain oncogenes, making them especially interesting for cancer research [100,101,102,103,104]. The quest to elucidate the biological role of G-quadruplexes has sparked the development of probes that can specifically detect them in cells. As a universal DNA intercalator with an extraordinary ‘turn-on’ response, TO has been shown to display increased fluorescence when interacting with G-quadruplexes, though not exclusively [26]. Hence, as in the case for sequence-targeting probes, tweaking the response of TO to selectively distinguish G-quadruplexes from other structures is essential.

Primary works by the group of Teulade-Fichou [105,106] exploited the interaction of TO with a telomeric G-quadruplex-forming sequence, 22AG, to create a fluorescent intercalator displacement (FID) assay for G-quadruplex binders [107,108]. The displacement of TO from the G-quadruplexes by competing compounds could be detected by following the decrease in TO’s fluorescence. Although the FID-based screen identified some novel G-quadruplex-binders [109] and even platinum anticancer drug candidates [110], such assays are often contested due to the promiscuity of free TO in binding to DNA. As seen in other works [109,111], the increased fluorescence of TO in the presence of G-quadruplexes is only moderately higher relative to dsDNA. The level of specificity is likely to be extremely influenced by the length and sequence of the examined targets, together with the chosen experimental conditions.

Thus, these studies indicate that chemical modifications of TO are needed to provide TO-based probes with selectivity toward G-quadruplexes (Figure 7b). The interest in selectively targeting G-quadruplexes has resulted in the discovery of many molecular ligands which exhibit high affinity towards G-quadruplexes [112]. Most commonly, such ligands consist of flat aromatic structures that can form stacking interactions with the planar G-quadruplexes. With addition of cationic elements, which can electrostatically interact with the negatively charged backbone, selectivity and tight binding to G-quadruplexes can be achieved. Several attempts at combining selective ligands of G-quadruplexes with TO have demonstrated the usefulness of this approach in sensing these unique structures. Monchaud [113] cleverly merged TO with a known high-affinity G-quadruplex binder, pyridodicarboxamide (PDC). This chimeric probe, called PDC-*M*-TO, was shown to be approximately 8-fold more selective in binding to the quadruplex-forming telomeric DNA 22AG than to random dsDNA, and slightly less for other G-quadruplex-forming sequences. Likewise, Wong [114] merged TO with a different G-quadruplex binder, cryptolepine, to afford a probe that exhibits 300-fold increased fluorescence upon recognizing quadruplexes of the *c-myc* promoter region and the telomeric sequence telo21. This probe also stained the nuclei of fixed cells, where it exhibited specific localization to areas presumed to contain quadruplex-forming ribosomal DNA. Suspected interactions with nuclear RNA were also examined, revealing that the probe exhibits increased fluorescence in the presence of RNA, although an even stronger one in response to G-quadruplex RNA. Chow [115], in a similar manner, generated a p-(dimethylamino)styryl-TO derivative that was able to exhibit 10-fold higher selectivity towards G-quadruplexes than to dsDNA. Fluorescent staining of cell nuclei by styryl-TO was shown to disappear after treatment with DNase but not with RNase, illustrating its selective binding to DNA. Notably, other styryl-TO probes prepared by Chow have produced opposite results, showing selectivity towards RNA but not towards DNA [116].

Over the years, many additional TO derivatives have been designed and tested for their selectivity towards G-quadruplexes. Some examples include cyclopentane-TO conjugates [117], isaindigotone derivatives for colorimetric detection [118], TO-BVMC conjugates [119], and various derivatives of styryl-TOs [120,121,122].

Although still in its infancy, imaging G-quadruplexes within cells using TO-based probes has a positive and promising future [123]. The proven applicability of TO-based molecular probes of G-quaduplexes so far serves as an important stepping-stone on the way to developing a specific, low-background and reliable method to study the biological role of these unique DNA conformations.

### 2.3. TO-Based Probes for Sensing Protein–DNA Interactions

**Figure 8:**
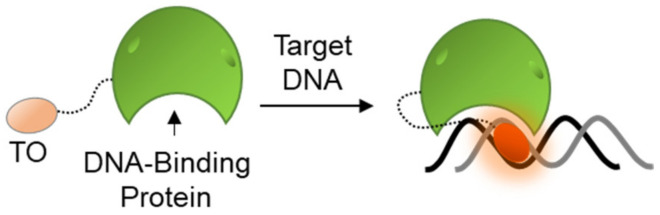
Sensing of DNA–protein interactions by TO-labeled DBPs. A chosen DBP is first covalently labeled with TO and then introduced to its target DNA. The proximity between the DNA and TO induces intercalation and a consequent increase in fluorescence.

The general tendency of TO to bind and fluorescently respond to nucleic acids has also lent itself to the research of protein–DNA interactions. Many of the cell’s functions are mediated by the activity of DNA binding proteins (DBPs), from DNA conformation-modulating histone proteins that control the folding and accessibility of chromosomes and genes, to a myriad of replication and transcription machineries that control the output of DNA-coded instructions. To follow the functions of such proteins, different studies utilized TO-labeled DBPs in order to identify protein–DNA complexes (Figure 8). Although covalently attached to proteins, TO’s fluorescence is still dependent on DNA binding and intercalation, thus essentially defining such probes as DNA sensors.

Woodbury conjugated TO to a peptidic zinc-finger binding domain of the glucocorticoid receptor [124]. Upon binding to a specific DNA recognition site of the receptor, TO exhibited increased fluorescence. The same approach was further applied to explore the thermodynamic nature of binding between more TO-labeled DBPs and their specific DNA sequences, as compared to non-specific sequences [125]. In another study, TO was used to label a reactive cysteine residue of the H3 histone protein in order to monitor the assemblies of nucleosomes [126]. Here, too, TO responded with a high fluorescence output upon binding the target of histone H3.

Despite the potential applicability of this approach, covalent attachment of TO onto a specific location in a protein is required, which poses several issues. Ensuring that the protein structure is not compromised by the labeling is crucial. Moreover, attachment of TO close to the DNA-binding domains, as is the case in the above examples, may induce promiscuity in DNA recognition of the probes, making them prone to reacting with random sequences, much like terminally TO-labeled DNA probes. It should also be taken into account, as explained in Section 5 below, that interactions between the attached TO and its neighboring amino acids might lead to increased fluorescence on its own, yielding background signals that can limit proper sensing.

## 3. TO in the Sensing of Small Molecules and Ions

Understandably, TO has made its mark in DNA and RNA research, owing to its remarkable ‘turn-on’ fluorescent properties. Nevertheless, controlling TO’s fluorescence through intercalation is just one approach available to fine-tune the optical responses of TO-based fluorescent sensors. On its own, TO can be chemically modified to exhibit increased fluorescence in response to small molecules or ions. For example, a study of a TO-derivative appended by two 17-atom-long aliphatic chains found that it exhibited selective increased fluorescence in the presence of ATP, relative to AMP, ADP, and other nucleoside triphosphates [127].

Another approach to sense small molecules concerns the dimerization and aggregation properties of TO, a phenomenon termed aggregation-induced emission (AIE) [128]. This approach, which has been exploited to develop the previously mentioned ECHO probes (Figure 6C) and the multicolor oligonucleotide-based probes (Figure 5), makes use of the tendency of TO to form aggregates in aqueous solutions [129], or when exposed to micelle-forming surfactants and macrocyclic cavitand molecules [130]. The formation of dimeric TO H-aggregates strongly affects the photophysical features of the dye. The most apparent effect results from excitonic interactions of TO dimers, which lead to a hypsochromic shift in TO’s absorption, peaking at ~470 nm, and an overall weaker yet red-shifted fluorescence above 600 nm. Aggregation of TO is a dynamic process: it readily shifts between monomeric, dimeric, and high-order aggregates according to the surrounding conditions. The apparent effect of aggregation on the absorption and fluorescence of TO has inspired studying the dye’s interaction with different host molecules, which can promote local aggregation states. Many of these studies have established TO as a reporter dye that can be utilized for recognizing and reporting small molecules in solution, harboring potential advantages for developing drug delivery devices, enzymatic assays, and diagnostic tools, among others.

**Figure 9:**
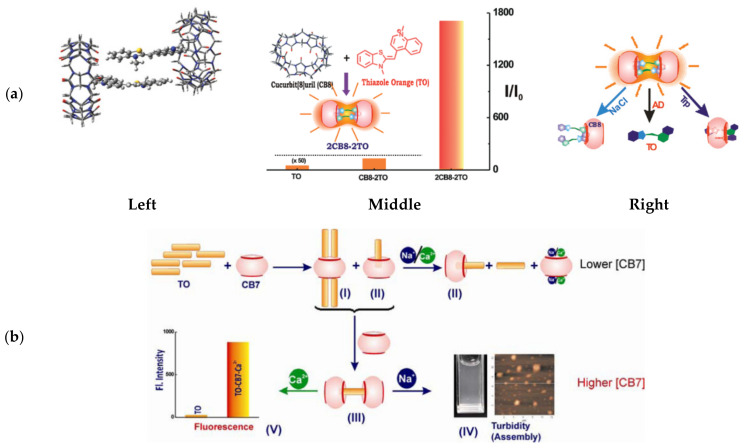
Examples of TO’s interactions with cucubit[n]uril host molecules. (**a**) Left: The structure of a formed 2:2 complex between CB8 and TO. Middle: Complex formation resulted in a 1700-fold increase in TO dimer fluorescence. Right: examples of nonemissive species generated in response to the presence of different analytes. Reproduced with permission from [133]. Copyright 2012 American Chemical Society. (**b**) Different CB7-TO complexes formed under different environments, displaying distinct responses to either sodium or calcium ions. Reproduced with permission from [134]. Copyright 2015 American Chemical Society.

Heyne [131] showed that TO interacts strongly with a negatively charged calix[4]arene sulfonate host molecule to form characteristic H-aggregates with a blue-shift in absorption. Fluorescent titration experiments suggested that three TO units align at the core opening of calix[4]arene through electrostatic interactions between the cationic dye and the host’s negative charges. Pang [132] identified complexes of TO with cucubit[n]uril (CBn) molecular hosts, specifically CB7 and CB8, and the formation of a linear supramolecular polymeric structure with the latter. Further work, done by Bhasikuttan [133], revealed that increasing concentrations of CB8 can form 2:2 complexes with TO, substantially stabilizing TO H-dimers in a rigid conformation (Figure 9a, left) that allows for an incredible 1700-fold increase in fluorescence at 605 nm (Figure 9a, middle).

Subjecting the 2CB8:2TO complexes to biologically relevant analytes, such as tryptophan or sodium ions, resulted in decreased fluorescence and distinct absorption shifts, indicating that different analytes are identified via distinct TO-release mechanisms (Figure 9a, right). A similar fluorescence response was observed from a complex formed between TO and a β-cyclodextrin derivative, which selectively disassembled in the presence of nanomolar concentrations of the neurotransmitter tyramine [135]. Subsequent research revealed that the host-guest interactions between TO and CB7 are dependent on the metal ions present in solution [134] (Figure 9b). Complexes of varying ratios of TO and CB7 were shown to exist in solution, depending on the host concentration; the 1CB7:4TO complexes are predominant at low concentrations and 2CB7:1TO takes over at higher concentrations. In response to the addition of metal ions, which compete over CB7 binding with TO, these complexes disassemble. Interestingly, a distinction could be made between Na^+^ and Ca^2+^ ions at high CB7 concentrations: Na^+^ ions yielded turbid particles, while Ca^2+^ ions retained solubility with increased H-dimer fluorescence. This observation was attributed to different ion-mediated assemblies being stabilized by the two ions, positioning TO host-guest probes as potential analytical tools for detecting metal ions.

**Figure 10:**
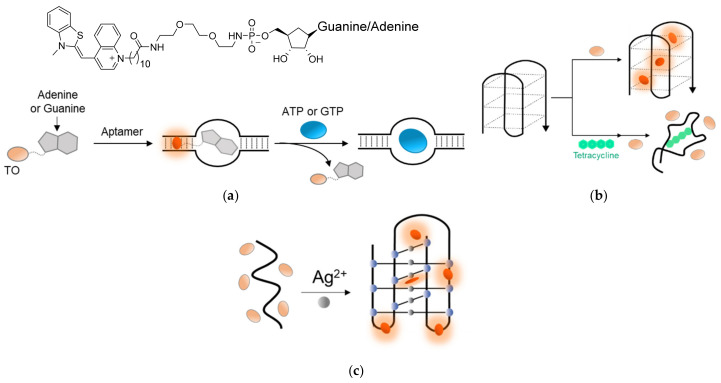
Sensing of ions and small molecules by coupled interactions of TO and nucleic acids. (**a**) The chemical structure of TO-AMP/GMP conjugates (top) that exhibit ‘turn-on’ fluorescence upon binding to ATP or GTP-specific RNA aptamers (bottom). ATP or GTP displace the conjugates from the aptamer, resulting in reduced fluorescence [136]; (**b**) Sensing of tetracycline by affinity changes towards TO resulting from conformational changes in its aptamer. When unbound to tetracycline, the aptamer adopts a G-quadruplex conformation with high affinity for TO. However, the binding of tetracycline yields a less structured conformation, thereby reducing the affinity of TO towards the aptamer and lowering its fluorescence [137]; (**c**) The binding of TO to an Ag^2+^-stabilized i-motif provides the means to detect Ag^2+^ ions by monitoring the increase in TO’s fluorescence [138].

TO’s outstanding interactions with DNA and RNA were also harnessed to detect ions and small molecules. Stojanovic [136] prepared TO-AMP and TO-GMP conjugates that exhibited an increased fluorescence in the presence of specific RNA aptamers for ATP or GTP as a result of the intercalation of TO with the RNA duplex (Figure 10a). Displacement experiments with free ATP and GTP resulted in decreased TO fluorescence by competing interactions with the aptamers. Zhang [137] took advantage of the interaction between TO and G-quadruplexes to track the binding of the antibiotic tetracycline to its aptamer (Figure 10b). The tetracycline-specific aptamer, which adopts a G-quadruplex conformation in solution, forms a highly fluorescent complex in the presence of TO. Once tetracycline is added, the aptamer shifts to a less structured conformation with reduced affinity towards TO, thus exhibiting lower fluorescence.

Induced TO fluorescence was also observed in the presence of DNA i-motifs [139,140], or i-DNAs, occasionally formed by cytosine-rich DNA sequences. As these motifs are also formed in the presence of silver ions, monitoring TO’s intercalation into an i-motif sequence could also be used to detect Ag^2+^ in solution (Figure 10c) [138]. These examples highlight the potential of TO to be applied as a reporter dye for a wide range of molecules and environmental conditions.

## 4. TO-Based Labeling of Genetically Fused Tags

### 4.1. TO Labeling of Protein Tags

The discovery of fluorescent proteins (FPs) revolutionized biology by enabling the monitoring of protein expression and dynamics, both at spatial and temporal resolutions [3]. FPs, such as the well-known GFP [141,142], can be genetically fused to a desired target protein, creating brightly fluorescent chimeras that serve in tracking and imaging of such targets in cells or even in whole organisms. Despite their great contribution to biological research, one limitation of FPs is their large size, which could disrupt the normal structure and function of the inspected proteins.

In an effort to image specific proteins in living cells while refraining from their fusion to large protein tags (i.e., FPs), an alternative tagging method emerged. With this approach the target proteins are expressed with an added short amino acid sequence-tag that can be selectively labeled by an externally added fluorescent probe [143,144], for example, the fluorescein arsenical helix binder (FlAsH) tetracysteine-specific probe and its analogues [145,146], the rhodamine-derived bisboronic acid (RhoBO) tetraserine-specific probe [147], and polyhistidine-specific probes [148,149]. Some of these probes exhibit ‘turn-on’ fluorescence responses upon binding to a tagged protein [145,146,147], thereby minimizing background fluorescence from unbound probes and, consequently, improving signal sensitivity.

**Figure 11:**
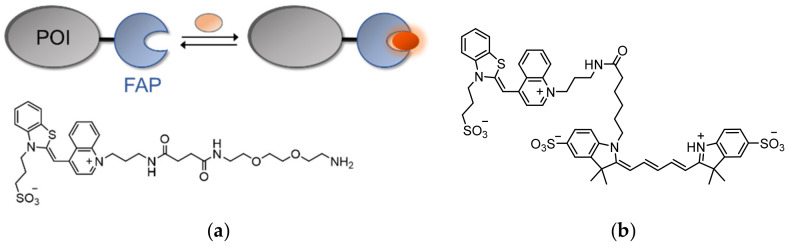
TO labeling of POIs fused to a fluorogen activating protein (FAP). (**a**) The operating principle of FAPs (top) and the structure of the TO-based fluorogen (bottom); (**b**) The structure of a pH-sensitive Cy5-linked TO fluoregen, which was used to track endocytosis of a membrane receptor fused to FAP. Internalization of the labeled target into acidic endosomes induced FRET between TO and the Cy5 analog [151].

Naturally, the low background emission of TO and its strong ‘turn-on’ fluorescence response make it an ideal candidate for labeling fusion tags in living cells. This was achieved by Waggoner [150], whose team introduced the technology of fluorogen-activating proteins (FAPs) by utilizing single-chain antibodies (scFvs) that can specifically bind TO and activate its fluorescence (Figure 11a). In order to avoid interactions with nucleic acids, TO was equipped with a negatively charged sulfonate group. In addition, TO was coupled to diethylene glycol diamine, which provided the fluorogen with water solubility (Figure 11a). Screening of scFV libraries and further optimization of a selected few led to the identification of a protein fragment (i.e., a FAP) that binds TO with a K_d_ value of 1.7 nM and induces a 2600-fold increase in TO’s fluorescence.

By expressing a FAP-modified platelet-derived growth factor receptor (PDGFR) in living cells and subjecting the cells to the TO-based fluorogen, the receptor’s membrane localization and its secretory pathway were visualized. As the fluorescence of FAPs depends on the binding of externally added TO, FAPs are more resistant to photobleaching effects, since photobleached molecules can be conveniently replaced by free TO in the solution. However, the reported TO-binding FAP is similar in size to GFP, maintaining the need to find fluorophore-activating modules that are at least as bright yet smaller. Overall, FAPs have been shown to be equally as effective as traditional fluorescent proteins in the labeling of cellular protein targets, with the added features of tunable and reversible fluorophore binding. In a subsequent work [151], TO-binding FAPs were shown to be applicable as pH biosensors. By coupling sulfonated TO with a pH-sensitive Cy5 analog (Figure 11b), a pH-controlled FRET-based FAP probe was created that exhibits higher FRET efficiency under acidic conditions. This property was successfully utilized to monitor the endocytosis of a FAP-tagged G-protein-coupled receptor (GPCR) in living cells. Such results paved the way for the development of next-generation fluorogen-based probes for protein imaging in biological systems [152,153,154].

### 4.2. TO Labeling of RNA Tags

Unlike FPs, which possess autonomous fluorescence, nucleic acids lack such fluorescent counterparts. Consequently, the detection and imaging of DNA and RNA inside living cells has proven to be challenging. In the quest to develop suitable fluorescent probes for these biomolecules, several methods have been described and thoroughly reviewed [155,156,157,158], many involving exogenous hybridization probes. This also includes previously mentioned examples of TO-based sensors, which can detect DNA and RNA, but also require careful considerations due to the possible demand for cell-delivery agents and unforeseen reactions within the cellular environment. In addition, these sensors are incomparable to FPs in terms of fluorescence intensity, versatility, and wide applicability.

In an attempt to fill this void, GFP has been utilized as part of a well-implemented technique for sensing RNA molecules in cells, by fusing it to a bacteriophage-derived MS2 protein [159,160]. MS2 is known to bind a specific 19-nucleotide stem-loop RNA structure with exceptionally high affinity. Co-expression of MS2-GFP and a RNA-of-interest containing the MS2 stem-loop binding site enables its endogenous non-covalent labeling. However, in using this two-component expression system, the relative expression levels of the MS2-GFP construct and the target RNA must be considered in order to avoid aggregate formation or high background fluorescence from unbound MS2-GFP. In addition, substantial target recognition can only be achieved by attaching at least six repeats of the MS2 recognition element to target RNAs [159], which may interfere with their native fold and function.

**Figure 12:**
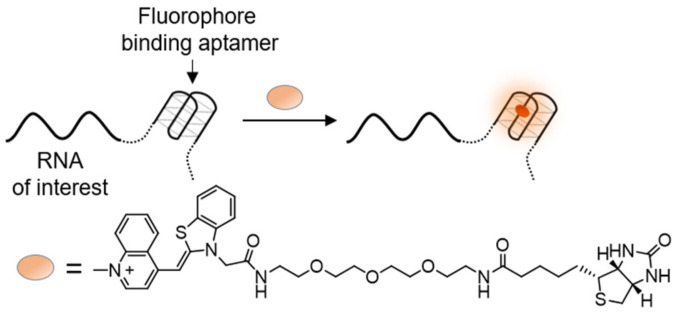
Labeling of target RNA molecules with TO-binding RNA Mango. Shown are the operating principle of RNA Mangos along with the chemical structure of TO-biotin.

In the RNA-of-interest, the stem-loop that binds MS2-GFP is essentially a RNA aptamer that can specifically bind the MS2 protein. Therefore, an interest arose in using small fluorescent dyes, which light up upon binding to specific RNA aptamers, as a possible replacement of the GFP fusion protein (Figure 12). Many such aptamer-dye pairs have been discovered [161,162,163,164], with the series of GFP-mimicking Spinach RNA aptamers being a prominent example. Spinach RNAs bind GFP-like fluorophores, increasing their fluorescence signal by 2000-fold and reaching to up to 80% of GFP’s intensity [165,166]. This, for the first time, provided RNA equivalents to FPs, which could then be attached to cellular RNA and be activated simply by adding a fluorogenic dye to be taken up by cells.

Following these studies, novel RNA aptamers, termed Mango RNAs, which can tightly bind and activate TO, were developed [167,168,169,170,171] (Figure 12). In order to prevent random intercalation of TO into dsDNA or dsRNA, the dye was conjugated to a biotin moiety. Detailed inspection of the interaction between TO-biotin and Mango RNA revealed binding of TO to a G-quadruplex in the center of Mango RNAs, yielding up to a 4000-fold increase in its fluorescence. RNA Mango systems improve upon their predecessor’s fluorogenic RNAs [172], owing to their compact size, brighter fluorescence, and the high contrast ability of TO. These features make RNA Mangos extremely valuable in the imaging of RNA molecules in cells, assisted by their non-disrupting attachment into untranslated regions of native RNA targets and an external addition of TO-biotin. Hence, RNA Mangos are comparable in their fluorescence intensity and possible applications to widely used fluorescent proteins, with the potential of having an equal contribution to RNA research. The utility of RNA Mangos was demonstrated by their ability to track both coding and non-coding RNAs [169,173] in fixed and live cells, as well as detect amplified pathogenic RNA [174].

## 5. TO in the Sensing of Proteins

Examples mentioned above of protein labeling (Figure 11), using TO-based probes, required that the protein of interest (POI) be fused to an engineered TO binding domain. Clearly, the need to genetically engineer the POI significantly complicates using such probes to study their biological activity, owing to the risk of accidentally affecting the POI’s expression, structure, or function. In addition, probes that can sense only engineered proteins are unsuitable for detecting disease biomarkers, such as proteins that are overexpressed in cancer cells. This excludes the possibility of using them in medical diagnosis.

**Figure 13:**
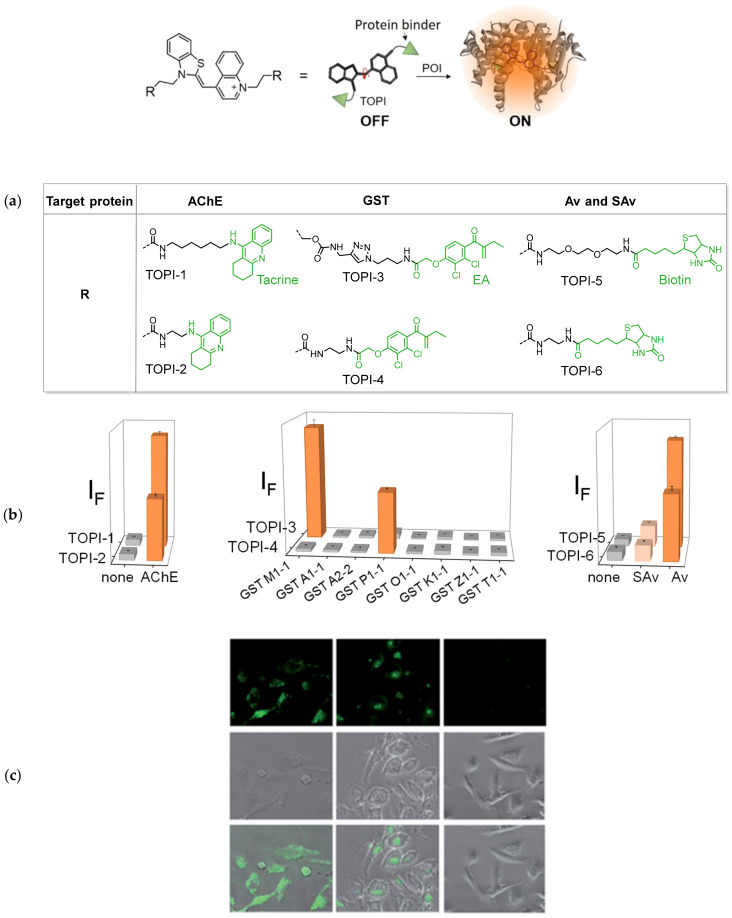
Protein sensing by TOPI probes. (**a**) The work mechanism of TOPI probes (top). TO is flanked on both ends by protein binders. Binding induces an interaction between TO and the POI’s surface, which restricts TO’s motion and yields increased fluorescence. Also shown (bottom) are the chemical structure of TOPI probes targeting different protein families; (**b**) The fluorescence responses of TOPIs to their protein targets; (**c**) Fluorescence (top), bright field (middle), and overlay (bottom) images of MDA-MB-231 cells overexpressing GST-P1-1, after incubation with TOPI-4 (left), TO (center), and TOPI-4 mixed with EA (right). Reproduced and adapted with permission from [175]. Copyright 2015 Royal Society of Chemistry.

To overcome these limitations, our group has developed TO-based sensors that can detect native (non-engineered) proteins. Specifically, we have shown that modifying TO with specific protein binders affords a new class of ‘turn-on’ probes, termed TO-based protein identifiers (TOPIs, Figure 13a), which can detect their protein targets with high affinity, selectivity, and a high S/N ratio [175]. The TOPI probes incorporate two identical protein binders tied by flexible linkers to TO through either of its aromatic components. Attached binders such as tacrine, ethacrynic amide (EA), or biotin afforded sensors that can selectively detect low nanomolar concentrations of acetylcholinesterase (AChE), glutathione-S-transferase (GST), and avidin (Av), respectively (Figure 13b). Although TO is essentially unresponsive to proteins, when incorporated as a component of TOPIs it lights up in response to protein targets, displaying between 7- and 55-fold increased fluorescence. Notably, TOPIs’ response was proven to be protein-exclusive, since most TOPIs displayed no fluorescence in the presence of DNA.

Perhaps most strikingly, certain TOPI probes displayed an isoform-selective fluorescent response (Figure 13b). For example, TOPIs bearing biotin groups exhibited larger increases in fluorescence in the presence of avidin rather than with the closely related streptavidin (SAv, Figure 13b, right). Similarly, TOPIs appended with two ethacrynic amides (EAs), contrasted by the structure and length of their linker units, exhibited remarkable selectivity toward one of several GST isozymes: GST-P1-1 or GST-M1-1 (Figure 13b, middle). These highly selective fluorescence responses were observed despite the ability of these TOPI probes to bind and inhibit a wide selection of GST isozymes. Hence, the most likely explanation of for such isoform-specific responses is that selective binding of TOPIs to their targets promotes non-specific interactions between the TO dye and the POI’s surface. As different isoforms possess distinct surface characteristics, each isoform creates a different molecular environment for TO, which distinctly affects the torsional restriction of the dye and the resulting fluorescence. The applicability of TOPIs was further demonstrated by using the GST-targeting probes in drug displacement assays, as well as in detecting GST and its interaction with an inhibitor (EA) in live cells (Figure 13c). Similar design principles were applied by Tan [176] to create monovalent TOPIs for detecting avidin, thrombin, and carbonic anhydrase II.

## 6. Conclusions and Outlook

This review summarizes research involving the use of TO as an essential component for creating ‘turn-on’ fluorescent probes for the sensing of a wide range of biomolecules and ions. Compared to traditional fluorescent dyes, the torsional control over the fluorescence of TO has the advantage of being internally embedded within the molecular structure of the dye. Therefore, TO-based fluorescent sensors do not necessarily require secondary auxiliary units to control their signal, in contrast with existing FRET-based probes. Additionally, the very low emission of TO in aqueous environments and the large increase in the dye’s fluorescence in response to restrictive interactions, sometimes reaching over 1000-fold, provides remarkable signal sensitivity through high S/N ratios. Finally, TO offers great versatility through chemical modifications that can be utilized to adjust its fluorescence response to different types of biomolecular targets.

The most detailed use of TO lies in the sensing of nucleic acids, for which TO’s intercalating nature has been well studied and understood. This has enabled TO to evolve from a simple DNA staining dye into a powerful tool that can be applied in several biochemical research methods, including those involving probes that have impressive sensitivity of up to a single nucleotide mismatch. However, the expanding application of TO to small-molecule and protein detection, as well as to sense specific nucleic acid conformations, leaves many unanswered questions regarding these less familiar interactions. Revealing the nature of these interactions is expected to lead to the development of even more accurate, sensitive, and selective TO-based probes.

In conclusion, we hope that this review will serve to publicize the advances made so far, and that it will inspire future endeavors that will contribute to our growing understanding of the mechanisms underlying TO-based probes and the ways to apply them in various areas of analytical biosciences.
